# Pathways to autism diagnosis in adulthood

**DOI:** 10.1186/s11689-025-09627-3

**Published:** 2025-07-01

**Authors:** Isabelle Dufour, Yohann Chiu, Sébastien Brodeur, Mireille Courteau, Josiane Courteau, Émilie Dubé, Alain Lesage, Éric Fombonne, Mélanie Couture

**Affiliations:** 1 École des Sciences Infirmières, Faculté de Médecine et des Sciences de la Santé (FMSS), Université de Sherbrooke, 3001, 12Th Avenue N., Sherbrooke, QC J1H 5N4 Canada; 2 Centre de Recherche sur le Vieillissement, Université de Sherbrooke, 1036 rue Belvédère Sud, Sherbrooke, QC J1H 4C4 Canada; 3 Centre de Recherche du Centre Hospitalier Universitaire de Sherbrooke (CRCHUS), Sherbrooke, QC Canada; 4 Département de Médecine de Famille et de Médecine d’urgence, Université de Sherbrooke, Sherbrooke, QC Canada; 5 Département de Psychiatrie et Neurosciences, Université Laval, Québec, QC Canada; 6 Centre de Recherche CERVO, Université Laval, Québec, QC Canada; 7 Institute of Psychiatry, Psychology & Neuroscience, King’s College London, London, UK; 8 École de Réadaptation, Université de Sherbrooke, Sherbrooke, QC Canada; 9 Département de Psychiatrie et d’addictologie, Université de Montréal, Montréal, QC Canada; 10 Department of Psychiatry, School of Medicine, Oregon Health & Science University, Portland, OR USA

**Keywords:** Autism spectrum disorder, Psychiatric disorder, Neurodevelopmental disorder, Mental healthcare, Healthcare use, State sequence analysis

## Abstract

**Background:**

This study explored Trajectories of Diagnoses (TDs) preceding a first diagnosis of autism in adulthood.

**Methods:**

This retrospective cohort study used health administrative data from Quebec, Canada, and included all adults with a first recorded diagnosis of autism between 2012 and 2017. A TDs was defined as a succession of medical records of psychiatric and/or neurodevelopmental conditions over time. These TDs were retrospectively analyzed from 2002 to 2017, using a state sequence analysis of diagnoses, in order: Autism, Intellectual or developmental disabilities (IDDs), Schizophrenia spectrum disorder (SSD), Bipolar Disorder (BD), Depressive Disorder (DD), Anxiety Disorder (AD), Attention-deficit/hyperactivity disorder (ADHD), and Other psychiatric and/or neurodevelopmental conditions.

**Results:**

The cohort included 2799 adults with a first recorded diagnosis of autism between 2012 and 2017. Several psychiatric and/or neurodevelopmental conditions were recorded since 2002, including AD (77.5%), DD (58.0%), SSD (49.4%), BD (48.3%), and IDDs (33.2%). Results revealed 5 distinct types of TDs. Types 1 (63.8%), 2 (17.6%) and 3 (6%) represented individuals in younger age groups with similar characteristics but with very different sequences of diagnoses, characterized by mixed diagnoses in type 1, SSD and AD in Type 2, and IDDs, DD, AD, and ADHD in type 3. Types 4 and 5 (9.0% and 3.6%), representing middle-aged/older groups, displayed distinctive TDs associated with high healthcare use, almost entirely associated with SSD (Type 4) and BD (Type 5).

**Conclusion:**

This study proposes a complementary examination of the multiple pathways to diagnosis experienced by adults, highlighting the need to address differential diagnosis and co-occurring psychiatric and neurodevelopmental conditions.

## Background

Autism is a complex neurodevelopmental condition characterized by early appearing social interactions and communications difficulties and restricted or repetitive sensory-motor behaviors. This condition was first recognized in the third edition of the Diagnostic and Statistical Manual of Mental Disorders (DSM-III) in 1980. At that time, infantile autism was considered rare, with a prevalence estimated between 3 to 7 in 10,000 children [1–3]. The DSM-III was revised in 1987 with a broadened concept of autism, and removing the requirement for onset before 30 months. The subsequent release of the DSM-IV in 1994 expanded the conceptualization of autism by introducing it as a "spectrum", including sub-categories defined as autistic disorder, Rett syndrome, childhood disintegrative disorder, Asperger’s disorder, and pervasive developmental disorder-not otherwise specified (PDD-NOS). In parallel, the 9th edition of the International Classification of Diseases (ICD-9), introduced in 1977, classified autism under “Pervasive Developmental Disorders” and was widely used in administrative databases. In 1992, the ICD-10 introduced more refined diagnostic categories—such as childhood autism, atypical autism, and Asperger's syndrome, supporting more precise classification of autism conditions. However, it was with the release of the DSM-5 in 2013 that the conceptualization of the "autism spectrum" underwent substantial changes. This revision acknowledged the broader range of manifestations of Autism Spectrum Disorder (ASD), now recognized as a more common yet heterogeneous condition, with a global prevalence estimated at around 1% to 1.5% [2, 4–7]. The spectrum concept recognizes the possibility of diagnosis in individuals whose challenges may emerge later in life, when social communication demands increase, particularly in adolescence or adulthood. The DSM-5 also proposes the use of specifiers in terms of levels of support needed, highlighting that autistic individuals can engage with the community to varying degrees [2, 4, 8].

Despite changes in diagnostic criteria and growing community awareness, many adults still face significant barriers to autism evaluation, including difficulty finding autism specialists for adults and long wait times, resulting in delayed diagnoses [9, 10]. Uncovering an autism diagnosis could benefit to adults who may otherwise perceive a flaw in themselves or view their social challenges as personal failure, experience disconnectedness and loneliness. This opens doors to potential educational and employment accommodations, joining support groups, sharing experiences, managing concurrent mental health issues like anxiety and depression, and help to reduce the risks of suicide [1, 10–14].

However, diagnosing autism in adulthood poses numerous challenges, including assessing individuals' developmental history, as parents or caregivers may be unavailable or provide less reliable information over time, the presence of coping and camouflaging strategies complicating the recognition of autism symptoms, and the influence of co-occurring psychiatric and/or neurodevelopmental conditions [2, 4, 9, 15–19].

A particular challenge arises when signs of autism overlap with psychiatric and/or neurodevelopmental conditions, which may partially mask core features of autism. Additionally, a diagnosis of autism could prevent the identification of true co-occurring conditions, emphasizing the need for a nuanced approach in differential diagnosis [19–24]. It is common for adults who receive their first autism diagnosis to have multiple psychiatric or neurodevelopmental conditions, which often serve as the primary reason for seeking medical care [15, 20, 25–27]. These conditions, including anxiety, depression, personality disorder, attention-deficit/hyperactivity disorder (ADHD), obsessive–compulsive disorder (OCD), bipolar disorder, schizophrenia spectrum disorder (SSD), and Intellectual or developmental disabilities (IDDs), are also associated with greater impairment in adaptive functioning and amplification of autistic symptoms [21, 22, 28]. Many factors may contribute to the co-occurrence of psychiatric and/or neurodevelopmental conditions, especially in adults with subthreshold or more subtle autistic traits. These factors include the challenges of living with autism, such as social isolation, overwhelming social demands, stressful life events (like changes in routines and environments), and experiences of bullying and exclusion. These challenges can lead to additional issues, most commonly depression and anxiety [11, 27, 29–32]. Other factors include shared pathophysiology or causes (such as anxiety or ADHD) and overlapping symptoms or diagnostic criteria [4, 11, 15, 23, 26, 33].

The pathway to autism diagnosis in adulthood remains poorly understood, particularly in the context of co-occurring psychiatric and/or neurodevelopmental conditions, which significantly and multifacetedly affect the misdiagnosis, missed diagnosis, or delayed diagnosis. Due to variations in study designs, methodological approaches, and the heterogeneity of populations, reported prevalences of co-occurring psychiatric and/or neurodevelopmental conditions in individuals with autism vary significantly. Furthermore, studies on lifetime psychiatric and/or neurodevelopmental diagnoses in autistic adults are scarce, and those focusing on adults rarely examine diagnoses made during adulthood [9, 21, 22, 34]. Hence, although each autistic adult experiences a unique pathway to diagnosis, there may be common patterns of mental healthcare service use, along with successive psychiatric and/or neurodevelopmental diagnoses preceding an autism diagnosis in adulthood. Exploring longitudinal pathways to autism diagnosis in a large population of adults can provide valuable insights into their shared experiences of mental health services and how these experiences evolve over time. A comprehensive understanding of pathways to autism diagnosis in adulthood can offer crucial guidance for mental health assessments, differential diagnosis, and facilitate the delivery of tailored services.

The objective of this study is to explore the pathways to autism diagnosis in adulthood from 2012 to 2017. This will be achieved by analyzing the trajectories of diagnoses (TDs) for psychiatric and/or neurodevelopmental conditions registered in the administrative databases of the public healthcare system, and having occurred from 2002 to 2017. This objective is twofold: (1) to establish a typology of sequences of diagnoses reported in medical records of adults before they receive a diagnosis of autism, and (2) to compare individual characteristics associated with each type of sequence.

## Methods

### Design and data sources

Data for this population-based retrospective cohort study were obtained from the provincial health insurance board (*Régie de l’assurance maladie du Québec*: RAMQ), which manages universal health insurance to Quebec residents, including coverage for physician and hospital services [35]. The RAMQ manages administrative health registers, including patients’ demographic information file, the hospital discharge register, and the medical services database (containing information from physicians’ claims for services provided in outpatient clinics, emergency and primary care clinics) within Quebec's public health system. The hospital discharge register contains information on the date and length of hospitalizations, the primary diagnosis, and up to 25 secondary diagnoses (ICD-9 before April 2006; ICD-10 thereafter). The patients’ demographic information file provides information on patients' age, sex (identified as male or female at birth), geographical location of residency, and date of death. The medical services register provides the date of service, the geographical location, and the diagnosis (ICD-9) associated with the service provided in the public healthcare system. The validity of diagnoses recorded in the RAMQ medical services claims and hospital discharge databases assessed for a variety of conditions have shown that SSD, bipolar disorder, and depression tend to have higher positive predictive values than anxiety disorders [35–37]. Patient data from these registers were linked to provide information on demographic, medical, and diagnostic information.

### Studied population

**Fig. 1:**
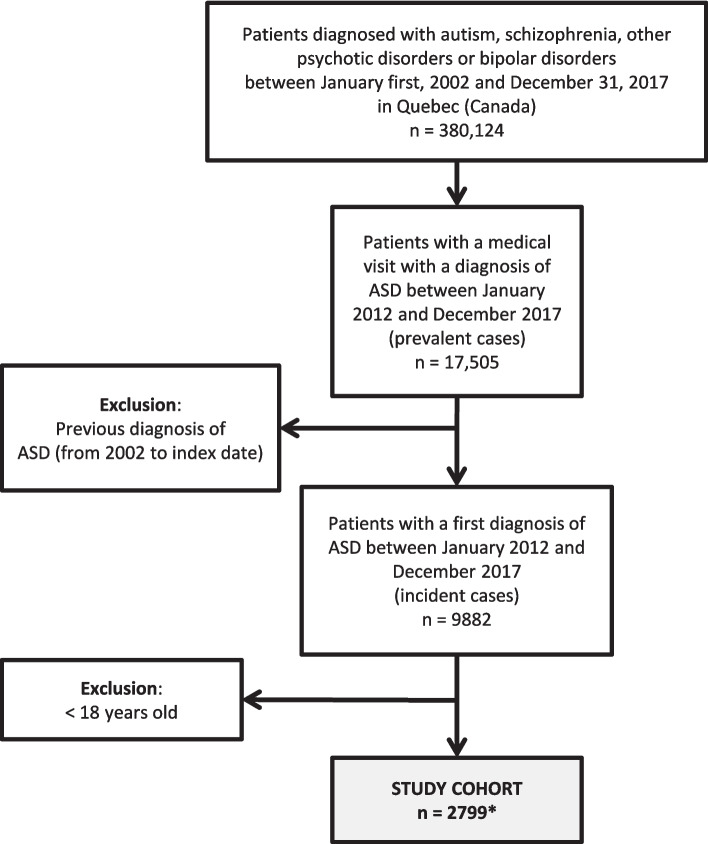
Study cohort flow diagram. *The study cohort consists of patients diagnosed with ASD in adulthood (*n* = 2799), representing 28% of all patients diagnosed with ASD (*n* = 9882) between January 2012 and December 2017

The cohort was extracted from a large database of 380,124 individuals living in Quebec (Canada) with registered diagnoses of autism spectrum disorder (ASD), psychotic disorder, or bipolar disorder, over a 16-year period (2002–2017). The study cohort included all adult individuals aged 18 and older, with a first diagnosis of autism (i.e., incident or first-recorded cases) documented in health administrative data between January 1st, 2012, and December 31st, 2017 (Fig. 1). The index date refers to the date of the first-recorded autism diagnosis during this 6-year period. Prevalent cases were defined as patients receiving a principal or secondary diagnosis of autism (ICD-9: 299; ICD-10: F84) during a hospitalization or during a medical visit (ICD-9: 299) [28]. To obtain first-recorded cases of autism only, we removed from the prevalent cases all patients with a previous diagnosis of autism between January 2002 and the index date (at least 10 years of clearance). As a first diagnosis may indicate suspected rather than established autism, our trajectory-based approach prioritized sensitivity to better capture adults entering the health system with an autism-related diagnosis. This case selection algorithm, previously validated in Canada and Quebec and applied in administrative data studies [28, 38], was identified as the most suitable among seven alternatives, with a sensitivity of 63%, specificity of 83%, and a c-statistic of 0.74."

### Variables

Characteristics of patients at the index date included: age (continuous and categorical, grouped as emerging, early, mid-adulthood, middle-aged, and seniors, adapted from Statistics Canada (2024) [39]: 18–24, 25–34, 35–49, 50–64, ≥ 65); sex (M, F); public prescription drug insurance plan status (see below); rurality, based on neighborhood characteristics (metropolitan: ≥ 100,000 inhabitants, small town: 10,000–100,000 inhabitants, rural: < 10,000 inhabitants); and a comorbidity index, from which we excluded mental health conditions. The selected index [40] is an adaptation of the 17 Charlson’s medical conditions [41, 42]. It was calculated over the two years preceding the first autism diagnosis to provide complementary information on individuals’ general physical health status. The public prescription drug insurance plan (PPDIP) admissibility status at the index date (as a proxy measure of low-income/unemployment status) includes three categories: not admissible (people with a private drug insurance plan); admissible and age ≥ 65 years with guaranteed income supplement (GIS) or being a recipient of last-resort financial assistance; or regular recipient.

In addition to examining the diagnostic trajectories themselves, the study also assessed the presence of at least one diagnosis of psychiatric and/or neurodevelopmental conditions recorded in health administrative data throughout the entire study period (from 2002 to the end of 2017). These conditions included: intellectual or developmental disabilities (IDDs), which include intellectual disability (ICD-9: 317–319; ICD-10: F70-F79) and specific delays in development (ICD-9: 315; ICD-10: F80-F83, F88, F89); schizophrenia spectrum disorder (SSD), including schizotypal disorder, acute schizophrenia-like psychotic disorder, and schizoaffective disorder (ICD-9: 295; ICD-10: F20, F21, F23.2, F25); bipolar disorder (types I and II) (ICD-9: 296; ICD-10: F30, F31); depressive disorder (ICD-9: 311, 300.4; ICD-10: F32, F33, F34.1); anxiety disorder (ICD-9: 300 except 300.4; ICD-10: F40-F42, F44, F45, F48); attention-deficit hyperactivity disorder, including all subcategories (hereafter referred to as ADHD) (ICD-9: 314; ICD-10: F90); Tourette syndrome (ICD-9: 307.2; ICD-10: F95.2); psychoses other than schizophrenia (ICD-9: 297, 298; ICD-10: F22-F24 (except F23.2), F28, F29); substance-related disorder (SRD) (ICD-9: 291, 292, 303, 304; ICD-10: F10-F16, F18, F19 (except F11.0, F12.0, F130, F140, F150, F160, F180, F190), F55); personality disorder (ICD-9: 301; ICD-10: F60-F62); and other mental, behavioral and neurodevelopmental conditions not mentioned above (ICD-9: 290–319; ICD-10: F01-99).

### Statistical analysis

Defined as a sequence of diagnoses over time, a Trajectory of Diagnoses (TDs) was measured over the whole 16-year available period (from January 1st, 2002-to 31st December 2017). To characterize TDs and define homogeneous groups, we used a state sequence analysis (SSA) approach, specifically developed to analyse sequential data [43–45]. For this study, a TDs was measured over 16 years, with “trimester” (3 periods of 28 days for each year) as the time unit. A state corresponds to a category of psychiatric and/or neurodevelopmental conditions registered in health administrative data, for each patient seeking mental healthcare (MHC) at a given time unit. To construct interpretable sequences, SSA requires each time unit to be assigned to a single state. As these states are not mutually exclusive, for each of the 69 trimesters, we defined the following 8 diagnosis states in priority order: 1. Autism; 2. Intellectual or developmental disabilities (IDDs); 3. SSD; 4. Bipolar disorder (types I and II); 5. Depressive disorder; 6. Anxiety disorder; 7. ADHD and/or Tourette syndrome (grouped based on clinical input due to their shared neurodevelopmental nature, early onset, and high co-occurrence rates of 50–60%) [46]; 8. Other mental, behavioral and neurodevelopmental diagnoses. The 9th state corresponds to no utilization of MHC service related to such diagnosis. After autism and IDDs, the priority order was established considering their relatively low prevalences and/or following the assumption that certain diagnoses (e.g., SSD, bipolar disorder) may dominate the clinical picture compared to others (e.g., anxiety), even if they co-occur. If a patient had more than one state during a given unit of time (e.g., a diagnosis of bipolar disorder and anxiety within the same trimester), the state with the highest priority, as listed above, was selected (bipolar disorder in this case). A matrix was then calculated containing the distance (proximity) between all pairs of patients’ TDs. The simple Hamming metric was used to measure the distance [43]. Based on this distance matrix, patients with similar TDs were grouped using agglomerative hierarchical cluster analysis with Ward’s method, which minimizes within-cluster variance while maximizing between-cluster variance [47]. The choice of Ward’s method was guided by best-practice recommendations in SSA [48], which recommend its use over all other algorithms. It also remains the most widely used clustering approach in SSA studies [44, 49]. The choice of the optimal number of groups or clusters was guided by statistical criteria (the partition with the highest relative loss of inertia [50], as well as interpretability and parsimony.

To interpret the typology of TDs, we used the State Distribution Plot (distribution of states [proportion] for each time unit point), and the Sequence Index Plot (individual’s sequence of diagnoses). Once each patient was classified into a specific type of TDs (or cluster), covariables between groups were compared using the usual descriptive statistics (Chi-2 test, Kruskal–Wallis test). As an additional descriptive summary of the TDs, we include bar charts showing the mean number of days spent in each state, stratified by TD typology and independent of the sequence structure. These charts offer a global view of the intensity of MHC use across and within TD types. Furthermore, because the priority order of diagnoses within a single day is less restrictive than across a trimester, these charts better reflect the overall distribution of diagnoses. The SSA was performed using the TraMineR package in R [43]. All other analyses were performed using SAS 9.4.

## Results

A total of 2799 individuals had a first diagnosis of autism registered in health administrative data during adulthood between 2012 and 2017 in Quebec (Fig. 1). Median age and quartiles show that half were diagnosed between 18 and 36 years old, and a quarter were diagnosed after 53 years old. For 45% of the cohort, the first diagnosis of autism occurred during a hospitalization, with 9.6% recorded as the primary diagnosis, followed by 22.2% in the Emergency Department (ED). In both ED and ambulatory settings, psychiatrists made 52% to 54% of the diagnoses, respectively (physician specialty information is not available for hospital discharge data). Notably, psychiatrists were more likely than GPs to diagnose autism in individuals with more complex psychiatric and/or neurodevelopmental profiles, as observed in Types 4 and 5. The sex ratio was 2 males for 1 female, and the majority (56.1%) were recipients of last-resort financial assistance or received a retirement pension with a guaranteed income supplement (Table 1). These patients received mental healthcare associated with several other psychiatric and/or neurodevelopmental conditions during the study period (2002–2017), including 33.2% with IDDs, ADHD/Tourette syndrome (20.4%), with only 3.7% of the cohort having a diagnosis of Tourette syndrome; and OCD (8.4%), as well as important proportions associated to anxiety disorder (77.5%), depressive disorder (58.0%), personality disorder (42.9%), SSD (49.4%), bipolar disorder (types I and II) (48.3%) and psychoses other than schizophrenia (53.5%) (Table 1). Between 2012 and 2017, more than 31% of the cohort had a registered diagnosis of autism in health administrative data only once, and almost 60% had a record of this diagnosis at least three times.

**Table 1:** Characteristics of the study cohort by the typology of Trajectories of Diagnoses (TDs) (*n* = 2799)

Characteristics	Total	Type 1	Type 2	Type 3	Type 4	Type 5	*P*-Value
	*n* = 2799	*n* = 1786 (63.8%)	*n* = 493 (17.6%)	*n* = 167 (6.0%)	*n* = 253 (9.0%)	*n* = 100 (3.6%)	
**MHC use**		Low to moderate	Low to high	High to moderate	Very high	High	
Age, median (IQR)	36 (24–53)	35 (23–52)	33 (24–49)	37 (24–52)	49 (36–57)	50 (33–58)	<.0001
Age group, n (%)							<.0001
18–24	707 (25.3)	507 (28.4)	139 (28.2)	43 (25.8)	8 (3.2)	10 (10.0)	
25–34	609 (21.8)	385 (21.6)	121 (24.5)	34 (20.4)	50 (19.8)	19 (19.0)	
35–49	635 (22.7)	384 (21.5)	115 (23.3)	41 (24.6)	75 (29.6)	20 (20.0)	
50–64	554 (19.8)	328 (18.4)	66 (13.4)	36 (21.6)	90 (35.6)	34 (34.0)	
≥ 65	294 (10.5)	182 (10.2)	52 (10.6)	13 (7.8)	30 (11.9)	17 (17.0)	
Sex, n (%)							0.0076
Female	912 (32.6)	581 (32.5)	157 (31.8)	50 (29.9)	75 (29.6)	49 (49.0)	
Male	1887 (67.4)	1205 (67.5)	336 (68.2)	117 (70.1)	178 (70.4)	51 (51.0)	
PPDIP status at index date^a^, n (%)							<.0001
Not admissible	599 (21.4)	416 (23.3)	104 (21.1)	23 (13.8)	31 (12.2)	25 (25.0)	
Admissible– regular	631 (22.5)	429 (24.0)	117 (23.7)	25 (15.0)	27 (10.7)	33 (33.0)	
Admissible– LRFA/GIS	1569 (56.1)	941 (52.7)	272 (55.2)	119 (71.3)	195 (77.1)	42 (42.0)	
Rurality at index date, n (%)^b^							0.6071
Metropolitan	2071 (77.8)	1303 (77.4)	364 (76.6)	128 (80.0)	197 (80.4)	79 (79.8)	
Small town	253 (9.5)	155 (9.2)	49 (10.3)	17 (10.6)	20 (8.2)	12 (12.1)	
Rural	338 (12.7)	225 (13.4)	62 (13.0)	15 (9.4)	28 (11.4)	8 (8.1)	
Care settings/providers for the ASD diagnosis at index date, n (%)							<.0001
During a hospitalization	1260 (45.0)	846 (47.4)	202 (40.3)	72 (43.1)	102 (40.3)	38 (38.0)	
Emergency department	620 (22.2)^d^	362 (20.3)	132 (26.8)	32 (19.2)	71 (28.1)	23 (23.0)	
Psychiatrist (ambulatory)	502 (17.9)	272 (15.2)	100 (20.3)	39 (23.4)	60 (23.7)	31 (31.0)	
GP/Other MD^c^ (ambulatory)	417 (14.9)	306 (17.1)	59 (12.0)	24 (14.4)	20 (7.9)	8 (8.0)	
Hospitalised at index date	1260 (45.0)	846 (47.4)	202 (40.3)	72 (43.1)	102 (40.3)	38 (38.0)	
Length of stay in days, median (IQR)	12 (4–31)	10 (4–25)	15 (5–37)	11 (4–26)	28 (9–84)	28 (14–62)	<.0001
ASD as the primary diagnosis, n (%)	121 (9.6)	74 (8.8)	27 (13.4)	11 (15.3)	9 (6.4)^e^	9 (6.4)^e^	<.0001
Other mental, behavioral, and neurodevelopmental disorders as the primary diagnosis, n (%)	597 (47.4)	333 (39.4)	122 (60.4)	33 (45.8)	81(79.4)	28 (73.7)	<.0001
Number of diagnosis of ASD, 2012–2017							<.0001
1	870 (31.1)	507 (28.4)	156 (31.6)	44 (36.4)	112 (44.3)	51 (51.0)	
2	257 (9.2)	150 (8.4)	48 (9.7)	19 (11.4)	32 (12.7)	8 (8.0)	
≥ 3	1672 (59.8)	1129 (63.2)	289 (58.7)	104 (62.3)	109 (43.1)	41 (41.0)	
Comorbidity index (≥ 1), n (%)	1047 (37.4)	648 (36.3)	187 (37.9)	62 (37.1)	109 (43.1)	41 (41.0)	0.2811
Psychiatric and/or neurodevelopmental conditions, 2002 to 2017 (at least one diagnosis), n (%)							
IDDs	929 (33.2)	584 (32.7)	169 (34.3)	90 (53.9)	67 (26.5)	19 (19.0)	<.0001
SSD	1384 (49.4)	681 (38.1)	296 (60.0)	82 (49.1)	253 (100)	72 (72.0)	<.0001
Bipolar disorder (types I and II)	1351 (48.3)	711 (39.8)	272 (55.2)	89 (53.3)	179 (70.8)	100 (100)	<.0001
Depressive disorder	1624 (58.0)	904 (50.6)	342 (69.4)	110 (65.9)	186 (73.5)	82 (82.0)	<.0001
Anxiety disorder (including OCD)	2169 (77.5)	1276 (71.4)	440 (89.2)	145 (86.8)	215 (85.0)	93 (93.0)	<.0001
OCD	236 (8.4)	119 (6.7)	59 (12.0)	23 (13.8)	20 (7.9)	15 (15.0)	<.0001
ADHD	571 (20.4)	363 (20.3)	120 (24.3)	56 (33.5)	17 (6.7)	15 (15.0)	<.0001
Tourette syndrome	104 (3.7)	55 (3.1)	22 (4.5)	17 (10.2)	5 (2.0)	5 (5.0)	<.0001
SRD	1062 (37.9)	565 (31.6)	238 (48.3)	52 (31.1)	143 (56.5)	64 (64.0)	<.0001
Personality disorder	1202 (42.9)	650 (36.4)	261 (52.9)	91 (54.5)	142 (56.1)	58 (58.0)	<.0001
Psychoses other than schizophrenia	1498 (53.5)	822 (46.0)	325 (65.9)	86 (51.5)	199 (78.7)	66 (66.0)	<.0001

*ADHD* attention‐deficit/hyperactivity disorder, *ASD* autism spectrum disorder, *GP* general practitioner, *IDDs* intellectual or developmental disabilities, *LRFA/GIS* last-resort financial assistance/guaranteed income supplement, *MHC* mental healthcare, *PPDIP* public prescription drug insurance plan, *SRD* substance-related disorders, *SSD* schizophrenia spectrum disorder

^a^PPDIP: public prescription drug insurance plan (as a proxy measure of low-income/unemployment status)

^b^Missing values: *n* = 137

^c^Other MD specialists represents less than 2% of cases

^d^Among the 620 patients diagnosed at ED, 208 were diagnosed by a psychiatrist, 323 by a GP, and 89 by another MD specialist

^e^Merged

**Fig. 2:**
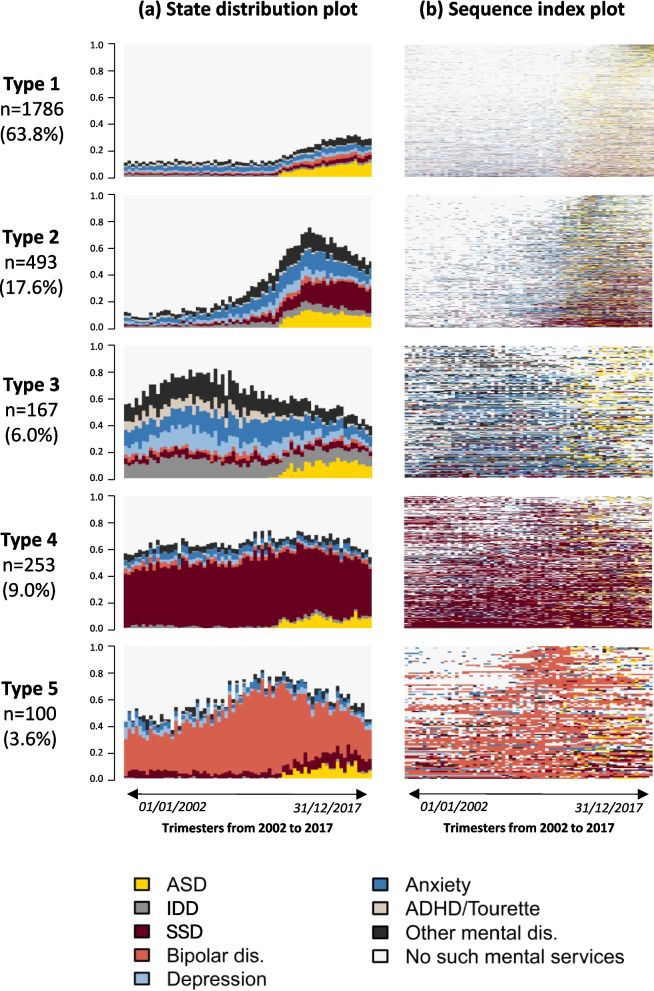
**a** State Distribution Plots and **b** Sequence Index Plot of the typology of Trajectories of Diagnoses (TDs). State Distribution Plots show the distribution of states (proportion) for each trimester of follow-up (69 semesters between 2002 and 2017) while the Sequence Index Plots show the individual’s sequence of registered diagnoses

**Fig. 3:**
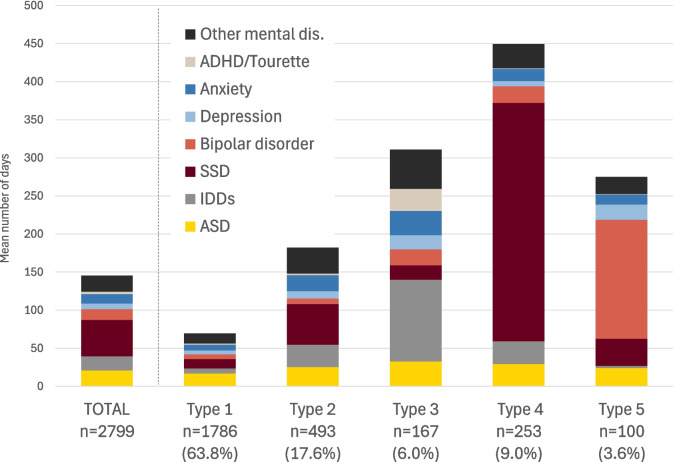
Mean number of days spent in each state by types of Trajectories of Diagnoses (TDs) during the 16-year follow-up period

The SSA revealed 5 distinct types of TDs over the study period (2002–2017) (Fig. 2). Nearly two-thirds of patients were associated to Type 1 (*n* = 1786, 63.8%), starting with low MHC use and displaying a slight increase in MHC use since 2012, almost entirely associated with autism diagnoses. Patients in Type 2 (*n* = 493, 17.6%) distinguish from Type 1 by a sharp increase of MHC utilization a few years before 2012, predominantly associated with anxiety disorders, SSD, and after the index date, with autism. Type 1 and 2 share similar individual characteristics, but some variations are observed in care settings/providers for the autism diagnosis (Table 1). Type 3 (*n* = 167, 6.0%) is characterized by very high MHC use with mixed diagnoses before the autism diagnosis and a significant decrease afterwards. Type 3 also shows the highest proportion of IDDs (53.9%), as well as ADHD (33.5%), OCD (13.8%) and Tourette syndrome (10.2%). Finally, Type 4 (*n* = 253, 9.0%) is characterized by a very high utilization of MHC throughout the period of interest, mainly for SSD (Figs. 2 and 3), while Type 5 (*n* = 100, 3.6%) is also characterized by a high utilization of MHC, but mainly for bipolar disorder. Every patient of Type 4 had at least one registered diagnosis of SSD, and every patient of Type 5 had at least one registered diagnosis of bipolar disorder between 2002 and 2017. However, more than 44% of patients in type 4 and 51% in type 5 had a registered diagnosis of autism only once. A diagnosis of ADHD was present in 6.7% of Type 4 patients, compared to 20% in the total cohort. Members of these two groups were also older, with median ages around 50 years. Furthermore, only 3.2% of patients in type 4 were aged 18–34, whereas almost half (49.0%) of members of type 5 were female (Figs. 2, 3, and Table 1).

## Discussion

### Delayed diagnosis of autism and psychiatric and/or neurodevelopmental co-occurring conditions

In line with previous studies, in our study, up to 28% of the individuals diagnosed for the first time between 2012 and 2017 were adults; the male-to-female ratio was 2:1, with half of the cohort receiving a first autism diagnosis at 36 years old and older (Fig. 1, Table 1) [1, 9].

Many factors can contribute to delayed diagnoses. A key factor is the age distribution of the study cohort: the median age of 36 years indicates that more than half of the individuals were born in 1981 or earlier, a period when autism was first recognized with the release of the DSM-III in 1980 and the ICD-9, adopted for administrative databases in Quebec in 1979 [1, 3, 8]. Consequently, most adults in the study cohort came of age at a time when signs of autism in children without IDDs were often overlooked, misdiagnosed, or never referred to mental health services. The expansion of diagnostic criteria toward a broader spectrum approach in the DSM-5 (2013) and the rise in community awareness have led to an increase in autism assessments [5, 9].

Other important reasons for delayed diagnoses have been proposed. Autistic individuals with more pronounced forms of autism are more likely to have been diagnosed in childhood, while those without IDDs and requiring lower levels of support may not face significant challenges until adulthood [2, 4, 15, 25]. A hypothesis suggesting a potential contributing cause of missed or delayed diagnoses is that the use of coping or camouflaging strategies (especially among females) may obscure the detection of autistic symptoms [4, 9, 17, 18], although questions have arisen in recent years regarding the foundations of this hypothesis [51, 52]. Several studies also suggest that females tend to exhibit less severe social communication challenges and fewer repetitive or stereotyped behaviors [6, 24]. That being said, many autistic individuals have reported poor past experiences with mental health services, prolonged treatments, and long waiting times, before obtaining an assessment for autism [4, 9, 10, 15].

As expected, most individuals in this study cohort were diagnosed with several psychiatric and/or neurodevelopmental conditions. IDDs, ADHD, and OCD fall within the range of pooled prevalences of recent meta-analyses on neurodevelopmental co-occurring conditions associated with autism [20–22, 27, 53]. However, the cohort's prevalence of anxiety, depressive and personality disorders, as well as SSD and bipolar disorder appears notably high compared to previous meta-analyses. However, an umbrella review [20] reported wide prevalence ranges for SSD (4–67%), depression (2.5–47.1%), bipolar disorder (4.4–37%), and anxiety (2.5–47.1%). Personality disorder prevalence varies from 6–12.6% [21, 54, 55], though rates of up to 60% in autistic adults have been reported [15]. Study design differences and reliance on health administrative data may contribute to overrepresentation of psychiatric conditions. In addition, the algorithm used to identify co-occurring conditions, based on at least one recorded diagnosis (favoured sensitivity over specificity), may overestimate prevalence. Nonetheless, a closer examination of the longitudinal patterns of diagnoses in the sequence index plots (Fig. 2b) supports the reliability of these conditions. For instance, repeated diagnoses of anxiety and neurodevelopmental conditions in Type 3, SSD in Type 4, and bipolar disorder in Type 5 suggest that reported rates reflect valid and often long-lasting conditions.

### Trajectories of diagnoses

Results from the state sequence analysis (SSA), a data-driven approach based on diagnoses received during MHC use over time, revealed highly variable pathways to autism diagnosis in adulthood. Five distinct types of TDs emerged (Figs. 2 and 3). The TDs Types 1 and 2, shared by the majority of the cohort (63.8% and 17.6%, respectively), exhibit similar individual characteristics, but very different sequences of psychiatric and/or neurodevelopmental diagnoses over time.

Types 1 and 2 also indicate that, before the increase in MHC seeking, anxiety disorders were likely predominant from adolescence or early adulthood, given the younger age groups in these types. It can be hypothesized that these types represent autistic individuals facing challenges of living with autism, resulting in additional disturbances [2, 4, 11, 32]. Several studies indicate that anxiety disorders are amongst the most common co-occurring conditions in youth with autism. Also, youth are estimated to frequently experience clinically significant anxiety symptoms in the range of 40 to 69%, commonly involving specific phobias, OCD, and social anxiety disorder, especially in those with IDDs [56–61]. Several hypotheses could explain the relatively low prior MHC use in Type 1. First, it is well established that many individuals with mental health conditions do not seek care, particularly younger individuals and males. Importantly, despite lower MHC use compared to other types, over 50% and 71% of individuals in Type 1 received at least one diagnosis of depression and anxiety, respectively, and more than 20% had a diagnosis of ADHD. Second, many may have initially sought care for physical health concerns, which frequently co-occur with autism and neurodevelopmental conditions. Nearly half of the index autism diagnoses in this group occurred during hospitalization, and only 48% of those hospitalizations were associated with mental, behavioral, and neurodevelopmental disorders, substantially lower than in other types. Nonetheless, over 63% of individuals in this group received three or more autism diagnoses over the study period, indicating subsequent referral and diagnostic confirmation. Upon closer examination of sequences of MHC use in Type 1, there is no noticeable change in healthcare-seeking patterns over time, except for autism. Conversely, in Type 2, after a peak around the index date, there is a decreasing trend in MHC use associated with other diagnoses alongside the autism diagnosis, except for SSD (affecting 60% of individuals). Studies have reported that individuals with intellectual disabilities are at an elevated risk of developing anxiety, mood, and psychotic disorders, which also frequently co-occurs with anxiety disorders [27, 62–64].

Type 3, representing 6% of the cohort, exhibits individual characteristics that barely differ from those in types 1 and 2 (Table 1), except for neurodevelopmental diagnoses, particularly IDDs (53.9%), ADHD (33.5%), OCD (13.8%), and Tourette syndrome (10.2%). Notably, members of Type 3 have likely experienced a prolonged history of high MHC use associated with anxiety disorders, ADHD and depressive disorders since childhood and adolescence, conditions known to frequently co-occur with autism [31, 32, 57]. The differential diagnosis of anxiety, depression or other conditions can be particularly challenging in individuals with IDDs, who may struggle to articulate their experiences, often resulting in misdiagnoses [9, 31, 56]. With the exception of IDDs, healthcare seeking gradually decreased alongside the initial diagnosis of autism. It can be hypothesized that once accurately diagnosed, autistic adults with IDDs in Type 3 may benefit from more tailored support and services.

Two additional distinct trajectories emerged from the SSA: Type 4 (9%), with a predominance of SSD, and Type 5 (3.6%), with a predominance of bipolar disorder, with about half of the individuals being female. Both types represent middle-aged to older individuals, with a median age of around 50 years at the index date. The co-occurrence of these disorders with autism is well-documented, with an increased prevalence of schizophrenia/psychotic disorders in males, and bipolar disorder in females [20, 22, 53, 65].

Although distinct conditions, autism, schizophrenia/psychotic, and bipolar disorders share overlapping clinical features, genetic patterns, and etiological risk factors, which could complicate differential diagnosis and delay the recognition of autism [26, 27, 53–55, 66, 67]. Clinicians may also prioritize early detection and intervention for psychotic and bipolar disorders, due to the potential impact of outcomes. For example, early detection of a first episode of psychosis is crucial for initiating appropriate treatment and significantly influences prognosis. However, distinguishing psychotic symptoms from non-psychotic conditions such as autism, which may present with similar features, can pose major challenges, even for expert clinicians [27, 68, 69]. Conditions requiring timely intervention, such as schizophrenia/psychotic and bipolar disorders, as well as major depressive disorder, might contribute to the delayed diagnosis of autism observed in Types 4 and 5 compared to other trajectories.

Beyond the influence of co-occurring SSD/psychotic and bipolar conditions in Types 4 and 5, the median age of 50 years signifies that more than half of these individuals were born in 1973 or earlier, well before the initial recognition of autism in the DSM-III. As a result, some of these adults may be regarded as a "lost generation" with a missed opportunity for autism assessment [15]. However, it is worth noting that 44–51% of patients in these types had only a single registered autism diagnosis, made later in life and despite a history of extensive mental healthcare use. This raises concerns about the validity of autism diagnoses among middle-aged adults with SSD or chronic psychotic and bipolar conditions. When autism is suspected, Quebec and Canadian guidelines (since 2012) recommend a comprehensive assessment involving medical history, physical examination, and developmental and behavioral evaluations [70–72]. For individuals with complex psychiatric profiles, diagnosing autism at a later age could reflect changes in diagnostic criteria, or represent"last resort"diagnoses of suspected autism, with referrals for comprehensive diagnostic assessment. However, these suspected diagnoses may not have been confirmed later [60, 73]. It is also notable that, across the five types of TDs, the proportion of individuals with only a single recorded ASD diagnosis, or potentially unconfirmed cases, tends to increase with age. These findings suggest possible overdiagnosis in middle-aged and older adults, particularly when presenting with a long history of SSD/psychotic or bipolar disorders and when developmental history is lacking. Prior research has shown that requiring multiple ASD diagnoses in administrative data improves the algorithm's specificity and positive predictive value, although it reduces its sensitivity [74–76].

### Differential diagnosis, misdiagnosis and overdiagnosis

Accurate diagnosis is incredibly challenging for clinicians, who often face difficult diagnostic differentiations within pressured or time-constrained contexts. Although expanded diagnostic criteria have increased sensitivity, especially for older adults without cognitive impairment, the ambiguous and heterogeneous phenotypic presentation of the autism spectrum, along with characteristics overlap with other neurodevelopmental or psychiatric conditions complicates the process, often resulting in misdiagnoses, delayed, or missed diagnoses of autism [4, 15, 23, 26, 27, 33, 77, 78]. Additionally, in clinical practice, assessment tools are often limited to a single diagnosis, making it challenging to evaluate co-occurring conditions. For example, the Autism Diagnostic Observation Schedule (ADOS) and the Autism Diagnostic Interview-Revised (ADI-R), widely used for diagnosing autism, focus solely on autism-specific traits and behaviors, overlooking co-occurring conditions. Furthermore, for older adults and women, more subtle or masked presentations of autism are frequently missed. This underscores the need for accurate diagnosis, comprehensive care, and treatments to address the full spectrum of needs in autistic individuals with co-occurring conditions [19, 23, 79–81].

Diagnostic overshadowing, particularly in autistic adults requiring minimal support, occurs when prominent symptoms of co-occurring psychiatric disorders, such as SSD/psychotic disorders, bipolar disorder, major depressive disorder, and personality disorder, mask signs of autism [16, 24, 33, 52, 82, 83]. This phenomenon may have contributed to the delayed diagnoses of autism observed in TDs Types 4 and 5. SSD and bipolar disorder, which typically emerge in adulthood (median onset: 25–33 years), contrast with conditions like ADHD, IDDs, and anxiety disorders (phobias, OCD, social anxiety, separation anxiety), which usually manifest in childhood and often persist over time [27, 32, 57, 59, 60, 66]. Members of Type 3, and a portion of Type 2, appear to have experienced these neurodevelopmental conditions more prominently. It is worth pointing out that, prior to the publication of the DSM-5, simultaneous diagnosis of ADHD and autism was not allowed [84, 85].

Several neurodevelopmental conditions share significant symptom overlap with autism, complicating diagnosis. Distinguishing autism from anxiety disorder and ADHD is particularly challenging, as behaviors like social withdrawal and repetitive actions, common in autism, can resemble those seen in social anxiety and OCD. While social challenges are not a core feature of ADHD or anxiety (unlike autism), individuals with these conditions often struggle with social interactions [32, 34, 60, 84, 86, 87]. Similarly, overlapping symptoms, such as social and communication challenges, as well as repetitive or atypical behaviors, can make it difficult to differentiate autism from later-onset conditions like SSD, bipolar disorder [53, 66], personality disorder [33, 88] and anxiety disorder [23, 57]. These similarities can lead to overdiagnosis or misdiagnosis of mental health conditions in autistic individuals. On the other hand, SSD and bipolar disorder are also frequently misdiagnosed, with SSD often mistaken for mood disorders, affective disorders, or nonspecific psychoses, while bipolar disorder is commonly misidentified as depression [89, 90].

Although misdiagnoses pose significant concerns, the issue of medical overdiagnoses has also gained attention in recent years [91–93]. Research has predominantly focused on conditions such as ADHD [85, 94–96], bipolar disorder [89, 97–99], as well as ASD [100–104]. A potential source of overdiagnosis in these conditions could be attributed to expanded diagnostic criteria and a lowered threshold.

### Autism diagnosis in adulthood

The expanded diagnostic criteria for autism have also resulted in a growing body of literature indicating a decrease in differences between individuals diagnosed with autism and those without [101, 105]. This trend poses additional challenges for clinicians in distinguishing between autism, neurotypical individuals, and other psychiatric or neurodevelopmental conditions [9, 15, 106]. However, the broader spectrum concept of autism offers benefits to adults with less complex needs, by providing them with opportunities to access the services and support they need [1, 10, 106].

The presentation of autism in adults varies due to several factors, including expectations of a formal diagnosis [10, 102, 107], the rise in self-diagnosis [108, 109], and the perception of stigma, with autism tending to be viewed more positively than psychiatric and personality disorders [78, 102]. Additionally, there are concerns about perceived misdiagnosis and a lack of trust in healthcare professionals, more often reported by females [10, 11, 24, 110]. On the other hand, individuals may also be not aware they have autism [1].

That being said, adults face significant obstacles before obtaining a formal diagnosis of autism. Previous psychiatric and/or neurodevelopmental diagnoses may represent distinct co-occurrent conditions resulting from living without an established autism diagnosis, which correlates with adverse experiences such as distress, disconnectedness, loneliness, and anxiety. These experiences may act as triggers for mental health difficulties and various psychiatric and/or neurodevelopmental conditions prior to receiving diagnosis of autism [1, 10, 11, 24]. Barriers to formal diagnosis in adults are commonly reported. These include difficulties to find adult autism specialists, poor access to mental health services and support, waiting times, and delays between the initial consultation and diagnosis, which are frequently reported in Canada [4, 9, 10, 109, 111–113]. It is important to note that diagnosing autism in adulthood generally has a positive impact, as it opens doors to tailored mental health for autistic people, helping improve social, educational and employment challenges, and reduce suicidal risk [1, 10–12, 14, 24, 27].

### Strengths and limitations

This study has several strengths. First, it operates within a real-world setting, using administrative data from an exhaustive cohort of 2799 individuals diagnosed with autism in adulthood. Second, to our knowledge, this study stands out as the first to offer a comprehensive view of psychiatric and neurodevelopmental diagnoses over an extended period. Finally, the use of SSA enables a straightforward examination of the most common types of trajectories of diagnoses on two levels: the diverse histories of psychiatric and neurodevelopmental conditions, and their evolution over time.

However, health administrative data has inherent limitations. Autism cases were identified through physician diagnoses from outpatient visits (ICD-9) or hospital discharges (ICD-10), where multiple, sometimes non-definitive, diagnoses are common, especially with complex co-occurring conditions. A diagnosis of autism recorded in administrative data does not necessarily indicate that it was confirmed through comprehensive clinical assessments. Additionally, autism may be underrepresented in public healthcare databases, as some individuals may have sought services (e.g., developmental or psychological) not covered by public insurance. As a result, some autism diagnoses may not represent true “incident” or first-recorded cases in health administrative data. However, it is important to highlight that autistic individuals often require significantly more healthcare due to the high prevalence of co-occurring conditions.

## Conclusion

These results highlight the challenge of identifying autism in adults, particularly for those with multiple prior or current psychiatric and/or neurodevelopmental diagnoses, potentially overlapping, exacerbating, or masking symptoms of autism, thereby contributing to the complexity of the differential diagnosis. As observed in Type 1 and during the early years of Type 2, the majority of the cohort, particularly those not diagnosed until early or mid-adulthood, made limited use of mental health services. This underscores the need to improve access to mental health services and ensure timely referral for multidisciplinary autism assessments. Types 3 to 5, characterized by high mental healthcare use, reveal additional diagnostic challenges: in early and mid-adulthood, differentiating autism from neurodevelopmental, anxiety, and depressive conditions; and in middle-aged and older adults (particularly females), where distinguishing autism from complex psychiatric presentations remains especially difficult. In this context, and given the broadening of diagnostic criteria and the considerable heterogeneity of autistic presentations, further research and clinical attention in adulthood is needed to better identify subtle or non-classic forms of autism, particularly when developmental histories are incomplete or unavailable. The reasons behind the oversight of autism until adulthood, a condition typically expected to manifest in childhood, remain poorly understood and are worth further investigation. This study proposes a complementary examination of the multiple, difficult pathways to diagnosis experienced by autistic adults in the real world, and how these experiences evolve over time.

## Data Availability

No datasets were generated or analysed during the current study.

## Abbreviations

ADHD: Attention-Deficit/Hyperactivity Disorder
ADOS: Autism Diagnostic Observation Schedule
ASD: Autism Spectrum Disorder
CDD: Childhood Disintegrative Disorder
DSM: Diagnostic and Statistical Manual of Mental Disorders
GIS: Guaranteed Income Supplement
ICD: International Classification of Diseases
IDDs: Intellectual or Developmental Disabilities
MHC: Mental Healthcare
OCD: Obsessive–Compulsive Disorder
PPDIP: Public Prescription Drug Insurance Plan
PDD-NOS: Pervasive Developmental Disorder-Not Otherwise Specified
RAMQ: Régie de l’assurance maladie du Québec
SRD: Substance-Related Disorders
SSA: State Sequence Analysis
SSD: Schizophrenia spectrum disorder
TDs: Trajectories of Diagnoses
